# Estimation of Ultrasound Echogenicity Map from B-Mode Images Using Convolutional Neural Network

**DOI:** 10.3390/s20174931

**Published:** 2020-08-31

**Authors:** Che-Chou Shen, Jui-En Yang

**Affiliations:** Department of Electrical Engineering, National Taiwan University of Science and Technology, Taipei 10607, Taiwan; m10707308@mail.ntust.edu.tw

**Keywords:** tissue echogenicity map, speckle reduction, convolutional neural network, image resolution, deconvolution

## Abstract

In ultrasound B-mode imaging, speckle noises decrease the accuracy of estimation of tissue echogenicity of imaged targets from the amplitude of the echo signals. In addition, since the granular size of the speckle pattern is affected by the point spread function (PSF) of the imaging system, the resolution of B-mode image remains limited, and the boundaries of tissue structures often become blurred. This study proposed a convolutional neural network (CNN) to remove speckle noises together with improvement of image spatial resolution to reconstruct ultrasound tissue echogenicity map. The CNN model is trained using in silico simulation dataset and tested with experimentally acquired images. Results indicate that the proposed CNN method can effectively eliminate the speckle noises in the background of the B-mode images while retaining the contours and edges of the tissue structures. The contrast and the contrast-to-noise ratio of the reconstructed echogenicity map increased from 0.22/2.72 to 0.33/44.14, and the lateral and axial resolutions also improved from 5.9/2.4 to 2.9/2.0, respectively. Compared with other post-processing filtering methods, the proposed CNN method provides better approximation to the original tissue echogenicity by completely removing speckle noises and improving the image resolution together with the capability for real-time implementation.

## 1. Introduction

Medical ultrasound imaging relies on the coherent summation of echoes from randomly distributed scatterers whose sizes are smaller than the acoustic wavelength. When the number of random scatterers within a sample volume is high enough, both the real and imaginary part of the stochastically interfered echoes will comply with normal distribution. Consequently, the amplitude of echoes becomes Rayleigh distributed and the resultant B-mode image suffers from granular patterns, which are called speckle noises. Note that, due to the nature of Rayleigh distribution, speckle noise is inherently multiplicative. The presence of speckle noise decreases the accuracy of estimation of tissue echogenicity from amplitude of echo signal. Moreover, B-mode imaging can be modeled as the convolution of the point spread function (PSF) of imaging system and the tissue echogenicity map with Rayleigh randomization [1]. Since it is impossible to have a Dirac-delta-like PSF, the detailed tissue boundaries inevitably become blurred in the B-mode image as compared to that in the tissue echogenicity map. The aforementioned speckle noise and the limited spatial resolution are the main factors that degrade the clinical diagnostic quality of ultrasound B-mode images.

Speckle reduction has been studied extensively in the context of ultrasound post-processing filters. However, state-of-the-art speckle-reduction algorithms such as non-local means filters (NLM) [2,3], anisotropic diffusion filter (ADF) [4,5,6], wavelet transforms [7] and bilateral filtering (BLF) [8,9] generally suffer from the degradation of spatial resolution and is unable to fully restore the original tissue echogenicity map. Deep learning has been successfully applied in the medical ultrasound for different tasks such as image segmentation [10,11], tumor localization [12], image enhancement [13,14], image reconstruction [15,16,17,18], beamforming [19,20] and also for speckle reduction [21]. In this study, we proposed a convolutional neural network (CNN) trained by simulated ultrasound images, to approximate the original echogenicity map by removing speckle noises from the input B-mode images together with enhancement of spatial resolution by deconvolution.

## 2. Methods

### 2.1. Training Dataset

**Figure 1:**
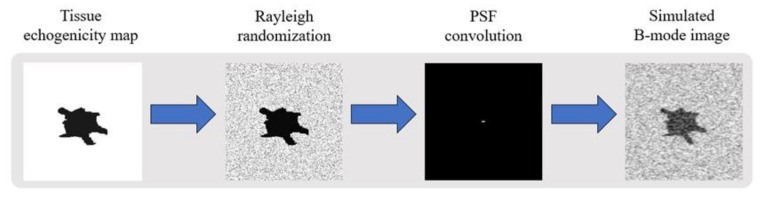
Flowchart of simulated ultrasound B-mode image.

**Figure 2:**
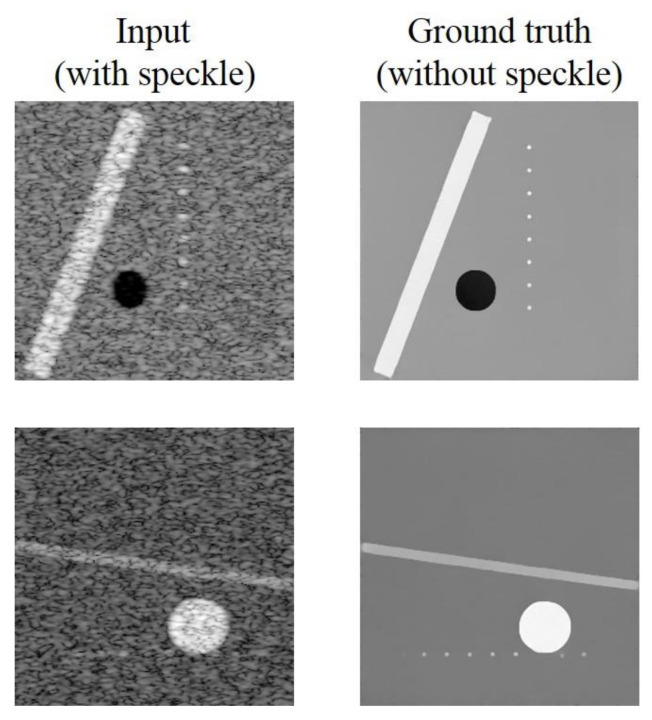
Training image pairs.

The training dataset is composed of simulated ultrasound images generated by MATLAB software using the following steps as shown in Figure 1 since original echogenicity maps of tissue are not clinically available. First, we generated three kinds of scattering structures in the echogenicity map of size 256 × 256, including circular contrast regions, hyperechoic rectangular regions and point-like reflectors. They simulated cysts, edges and wires, respectively. Sizes and positions of them are randomly assigned. After that, a complex random image is generated with normal distributed real and imaginary parts and then multiplied with the original echogenicity map to produce the reflectivity amplitude. Lastly, we defined a PSF and convolve it with the reflectivity amplitude to obtain the ultrasonic B-mode image with speckle noises. Here, the PSF is modeled as a two-dimensional Gaussian with specified size in both axial and lateral directions. The simulated B-mode images and the corresponding original echogenicity maps serve as the input and the ground truth pairs for network training as shown in Figure 2.

Twenty-eight possible combinations of echogenicity magnitude of the three kinds of scattering structures are listed in Table 1. The echogenicity magnitude is represented in decibel (dB) relative to the background level. There are totally 9800 pairs of image with 350 pairs for each combination. 70 % of the dataset are used for training with the rest 30% for validation.

### 2.2. Convolutional Neural Network

**Figure 3:**
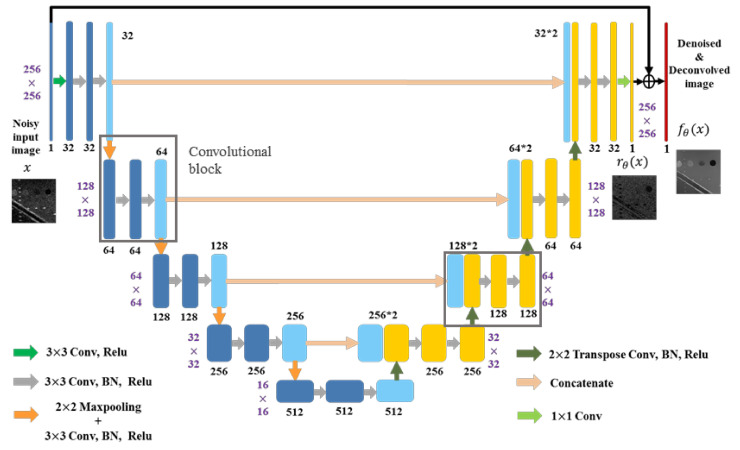
The proposed network architecture.

For the speckle-removal task in this study, an Unet-type convolutional neural network was used [22]. The network is composed of an encoding path and a decoding path, as shown in Figure 3. First, all noisy training samples are processed by five convolutional blocks in the encoding path. Except for the first block, each block includes a maxpooling layer and three 3 × 3 convolutional layers followed by batch normalization (BN) and rectified linear unit (Relu). Note that the number of feature maps changes from 1 to 512 in the encoding path. Then, a decoding path is cascaded, which contains a 2 × 2 transpose convolutional layer and two convolutional layers in each convolutional block. The number of feature maps reduces by half after each block in the decoding path. In addition, the concatenated layers are connected and input the feature maps from the encoding path to equal-sized maps in the decoding path. Lastly, we applied residual learning [23] by adding the output of a 1 × 1 convolution $r_{\theta }\left(x\right)$ and the input noisy image x to get the reconstructed echogenicity map $f_{\theta }\left(x\right)$.

For the network training, we applied and compared two training options in this study, including mixed loss function and the general mean square error (MSE) loss function. The sensitivity of structural similarity (SSIM) between images is considered to be very similar to the human visual system and thus the proposed mixed loss function here includes multi-scale structural similarity (MS-SSIM) and mean absolute error as suggested on task of image restoration and speckle reduction [24]. Specifically, the mixed loss function is defined as $L_{mixed}=L_{l_{1}}+\beta L_{MS-SSIM}$, we heuristically set β to 300 to equalize the contributions of each loss function. Training are done with the batch size of 8 and the epochs of 30. The optimizer for mixed loss function and MSE loss function is Adam and Step decay, respectively. The hyperparameter settings for the CNN network are listed in Table 2, where minimum number of filters stands for the filter number of the first convolutional block in the encoding path and the last convolutional block in the decoding path of the CNN network. Note that the number of filters in other convolutional blocks will change in proportion to the predefined minimum number of filters. We use different convolutional filter sizes, the minimum number of filters and the initial learning rate to test the effect on speckle reduction and deconvolution effects of different models.

### 2.3. Quantitative Analysis

**Figure 4:**
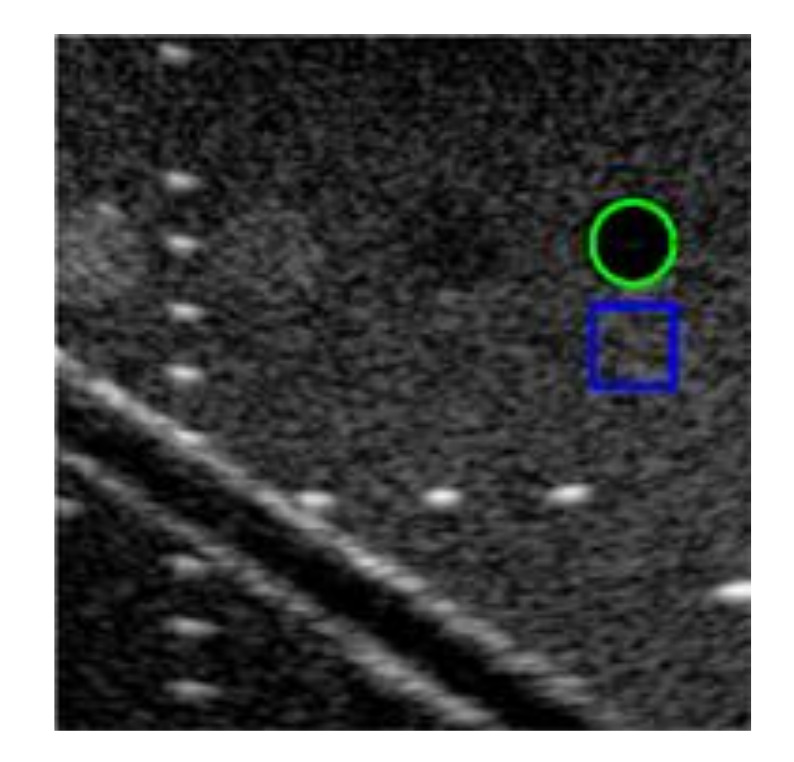
The test image with ROIs of target cyst (green) and background (blue) for quantitative analysis.

We use an ultrasound image of a commercial phantom (Mini-Doppler Flow 404, Gammex, USA) acquired by a linear array with center frequency of 6.4 MHz in Prodigy ultrasound platform (S-Sharp, Taiwan) to test our models. The reconstruction quality of the test image is measured using the image contrast (CR), contrast-to-noise ratio (CNR) and Full width at half maximum (FWHM) of wire reflectors in unit of pixels. The region-of-interest (ROIs) of target cyst (green) and background (blue) for CR and CNR calculation are shown in Figure 4. Since the test image has been log-compressed, we directly use the difference of mean magnitude between the two ROIs to calculate the CR. In order to further confirm the effect of the models under different parameter settings, we also test MSE, SSIM, Peak signal-to-noise ratio (PSNR) and mutual information (MI) on 10 simulated images outside the training dataset. These image metrics can be formulated as the formulas from (1) to (5). In (3), *X* is the original echogenicity map, *Y* is the reconstructed echogenicity map, *m* and *n* are the length and width of the image. In (4), $MAX_{I}$ is the maximum value of pixel, if each sampling point is represented by 8 bits, then this value is 255. In (5), $P_{X}(k)$, $P_{Y}(k)$ and $P_{XY}(k)$ are respectively the marginal probability distribution functions and the joint probability function of the original echogenicity map and the reconstructed echogenicity map when the pixel value is *k*.

(1) $$\mathrm{CR}=\left|\mu_{cyst}-\mu_{background}\right|$$

(2) $$\mathrm{CNR}=\frac{\left|\mu_{cyst}-\mu_{background}\right|}{\sqrt{\sigma_{cyst}{}^{2}+\sigma_{background}{}^{2}}}$$

(3) $$\mathrm{MSE}=\frac{1}{mn}\sum_{i=1}^{m}\sum_{j=1}^{n}{\left[X(i,j)-Y(i,j)\right]}^{2}$$

(4) $$\mathrm{PSNR}=10\log_{10}(\frac{MAX_{I}{}^{2}}{MSE})$$

(5) $$\mathrm{MI}=\sum_{k=0}^{255}P_{XY}(k)\cdot \log\frac{P_{XY}(k)}{P_{X}(k)\cdot P_{Y}(k)}$$

## 3. Results

Four comparisons among different settings of training data parameter and neural network hyperparameters are performed in this study.

### 3.1. PSF Size

As mentioned in the Section 1, the generation of speckle pattern in B-mode images involves convolution with the PSF of the imaging system. Here, we consider the impact of the PSF size on the reconstruction of echogenicity map. Therefore, we first test the impact of the PSF size definition on the reconstructed results. Note that the PSF of test image can be estimated by the following steps. First, we choose several wire regions in the test image and find their position coordinates where reflectivity magnitude is 6 dB lower than the peak magnitude in each wire region. After that, we calculate the distances between the endpoints in both axial and lateral directions of the coordinate. Finally, the calculated distances of all wire regions are averaged to obtain the estimations of PSF size.

**Figure 5:**
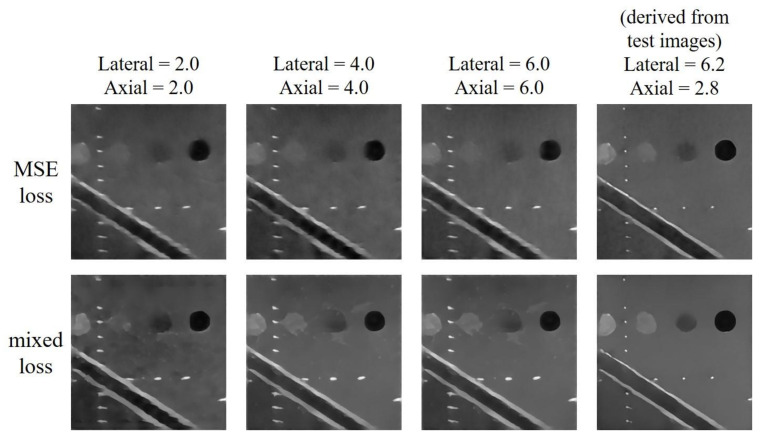
Reconstructed echogenicity maps of the two training options using training dataset of different size of point spread function (PSF).

For the test image in Figure 4, the lateral and axial size of PSF of the test dataset are respectively estimated to be 6.2 and 2.8 pixels. The reconstructed results are shown in Figure 5. For this test, the minimum number of filters and the filter sizes are respectively 32 and 3 × 3 for both training options. And the initial learning rate for the MSE loss model is 0.001 while that for the mixed loss model is 0.00035. The results indicate that, when the PSF size of the training dataset does not match that of test dataset in either the axial or lateral direction, the reconstructed echogenicity maps of the both training options become blurred with unsmooth background and unclear boundaries of cysts and edges as shown in the first three columns of Figure 5. Nonetheless, when the estimated PSF is utilized to construct the dataset for training the CNN model, the reconstructed echogenicity maps in the last column of Figure 5 shows finer scattering structures and cleaner background.

Quantitative analysis of the two training options under models of different PSF size are presented in Table 3. We highlight the best value for each metric in bold. All the metrics are improved as the PSF size matches between the training and the test image. Therefore, for the reconstruction task discussed in this paper, the PSF size of the training dataset used by the CNN must be set by the estimated value of the test image to obtain an ideal reconstruction of echogenicity maps. Since the model with mixed loss function performs better than MSE model on all metrics except image resolution, we will only consider the mixed loss training option in the following tests.

### 3.2. Minimum Number of Convolutional Filters

**Figure 6:**
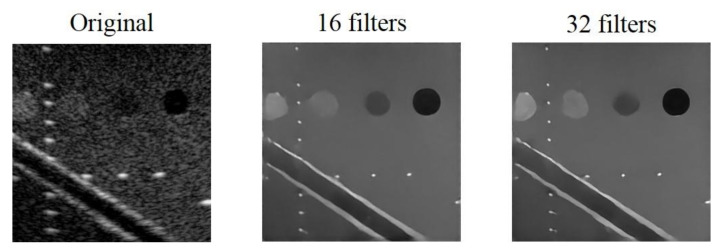
Original B-mode image and the reconstructed echogenicity maps of the mixed loss model when the minimum number of filters are 16 and 32.

We set 16 and 32 as the minimum number of convolutional filters for testing while the initial learning rate and the filter size of the model are 0.00035 and 3 × 3, respectively. The reconstructed results are shown in Figure 6. The difference of the results using the two different minimum number of filters are not visually significant. Nonetheless, compared with the original B-mode image, the effects of speckle reduction and deconvolution are obvious in both settings of minimum number of filters.

Quantitative analysis of the reconstructed echogenicity maps under different minimum number of filters are listed in Table 4. We highlight the best value for each metric in bold. In general, almost all the metrics improves when the minimum filter number of filters is 32, especially for CNR. Note that, when 16 is used as the minimum number of filters, the lower CNR means that the reconstructed echogenicity maps are less uniform in the cyst and background regions. In terms of image resolution, although the model has slightly worse FWHM values when the minimum number of filters is 32, the difference for them is visually indistinguishable in the B-mode images. Therefore, we choose to set the minimum number of filters to 32 in the following of this study.

### 3.3. Convolutional Filter Size

**Figure 7:**
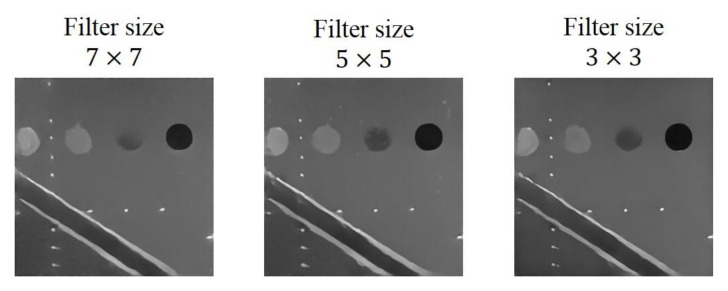
Reconstructed echogenicity maps of the mixed loss model with different filter size.

Note that, in Figure 3, all the convolutional layers are constructed with filter kernel of size 3 × 3. For comparison, filter kernels of size 5 × 5 and 7 × 7 are also used for testing their effect of reconstruction. The initial learning rate and the minimum number of filters of the model are 0.00035 and 32, respectively. The reconstructed results with different filter sizes are shown in Figure 7. As filter size decreases, the contour of the cysts become shaper and speckle noises in the background can also be eliminated more effectively.

Quantitative analysis of the reconstructed echogenicity maps using the three different filter size are included in Table 5. The best value for each metric is highlighted in bold. The CR and the CNR both improve as the filters become smaller. For the other metrics, the model with filter size of 3 × 3 can also obtain the best values. In contrast, the image resolution of the reconstructed echogenicity maps with the three filter sizes shows no obvious difference.

Note that the total number of parameters and the corresponding computational complexity in the CNN model increases with the filter size. A large filter size may hinder the CNN model from real-time implementation in practical applications. Therefore, after considering both aspects of reconstruction performance and implementation efficiency, we choose to set the filter size to 3 × 3 in this study.

### 3.4. Initial Learning Rate (Initial lr)

According to the sensitivity of the mixed loss training option to the learning rate, we use a specific learning rate range for testing.

**Figure 8:**
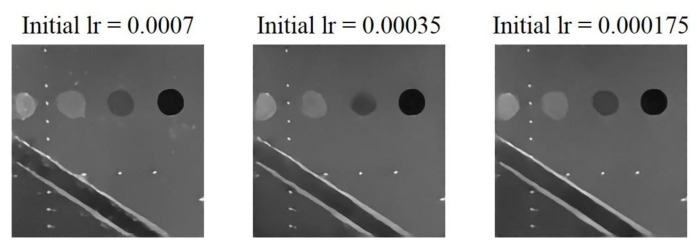
Reconstructed echogenicity maps of the mixed loss model with different initial learning rate.

As the results shown in Figure 8, the clarity of the three scattering structures (i.e., cyst, wire and edge) are all improved when initial learning rate is smaller than 0.0007. For the learning rates of 0.00035 and 0.000175, their reconstruction results are very similar to each other and both exhibit no obvious artifact in the background. The model with initial learning rate of 0.000175 can provide visually finer reconstruction of scattering structures. Quantitative analysis of the reconstructed echogenicity maps is listed in Table 6 and the best value for each metric is highlighted in bold. Although the reconstructed echogenicity map has a finer visual performance in edge preservation when the initial learning rate is 0.000175, the performances of all the metrics can be maintained or even better when the initial learning rate is 0.00035. As for image resolution, even though the reconstructed echogenicity map has the best resolution in both directions when the initial learning rate is 0.000175, the difference between it and the model with initial learning rate of 0.00035 is minor. Meanwhile, the other metrics such as CNR, PSNR and MI consistently get improved with the initial learning rate of 0.00035. Therefore, we choose the model with the initial learning rate of 0.00035. Note that, when the initial learning rate is too high or too low, the model will not be able to converge to the global minimum of the loss function for a specific task within a fixed training period, resulting in sub-optimal reconstruction results.

Due to the aforementioned results, it appears that the proposed method has the best performance when the CNN model adopted the mixed loss function and trained with the measured PSF size. In addition, other hyperparameters such as the filter size, the minimum number of filters and the initial learning rate have also been optimized to be 3 × 3, 32 and 0.00035, respectively. Using NVIDIA GeForce GTX 1080 GPU with 8 GB of memory, the training time for the proposed CNN model is approximately 2 h.

### 3.5. Comparison with Other De-Speckling Methods

We compare the proposed CNN model with mixed loss function and optimized settings of hyperparameters to other image de-speckling methods including the NLM, ADF and the BLF methods for their performance in speckle reduction.

**Figure 9:**
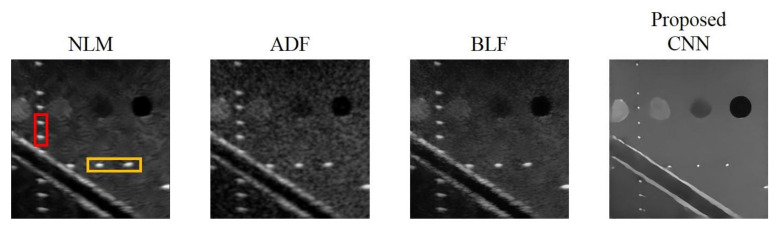
Reconstructed echogenicity maps of the four de-speckling techniques.

**Figure 10:**
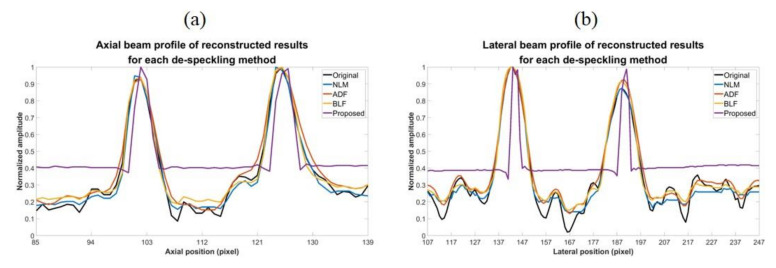
(**a**) Axial and (**b**) lateral profiles of the wire targets respectively in the red box and the yellow box of the reconstructed images for each de-speckling method.

Reconstructed results are shown in Figure 9 and the lateral and axial profiles of the wire targets in the yellow and red boxes are also demonstrated in Figure 10 for each method. All the three post-processing filtering methods can reduce speckle noises to some extent by suppressing the fluctuation of the background amplitude value. Note that the NLM and BLF methods appear to provide more noticeable speckle reduction while the reconstructed echogenicity map using the ADF method is not much different from the original B-mode image.

Quantitative analysis of the reconstructed results is listed in Table 7. Though all the NLM, ADF and BLF methods can maintain CR and provide higher CNR, their image resolution does not show much improvement in both directions. For the BLF method, the FWHM of the wire targets even degrades from 5.9 to 7.1 and from 2.4 to 3.1, respectively in the lateral and axial directions. It should be noted that, however, the spatial resolution of all the NLM, ADF and BLF methods can be enhanced by an additional deconvolution process but at the possible cost of counteracting their speckle smoothness. For the other metrics, the NLM and BLF methods can reduce MSE and enhance SSIM and PSNR, but also make MI worse. Compared to these post-processing techniques, the proposed CNN method can simultaneously remove the speckle pattern and retain the boundary of the vessel structures and cysts. Additionally, the CR and CNR of the reconstructed echogenicity map also significantly improve and the wire targets can be clearly identified as point-like reflectors.

Overall, the proposed CNN method provides much better performance for the task of reconstruction of echogenicity map. For example, the lateral and axial FWHM width improves to 2.9 and 2.0, respectively. Finally, in order to compare execution speed, we used the i7-4820K CPU with a memory size of 32 GB and MATLAB software (MathWorks, Natick, MA, USA) to obtain the total time required for the four methods to process 500 images with size of 256 × 256 pixels. From the results, it can be found that the proposed CNN method has a comparable or even superior processing speed of a single image to that of other de-speckling methods, indicating that the CNN method can also meet the real-time requirements of ultrasound scanning.

## 4. Discussions and Conclusions

In this study, a CNN model was proposed to reconstruct the tissue echogenicity maps in ultrasound imaging by removing speckle noises together with enhanced spatial resolution. We applied two kinds of training options and compared their results under different restriction of neural network hyperparameters. We found that the reconstruction task can be optimized only if the PSF size of training dataset is set according to that of test image. Moreover, the model trained by the mixed loss function can reconstruct clearer echogenicity maps than using MSE loss. From the results, the proposed method can reduce the difficult of reading ultrasound images by effectively eliminating speckle noises in the test image, while maintaining the structural contours and highlighting the boundaries of tissue. The CR and the CNR of the reconstructed echogenicity map increase from 0.22/2.72 to 0.33/44.14 and the lateral and axial FWHM also improve from 5.9/2.4 to 2.9/2.0, respectively. Compared with other post-processing filtering methods, the proposed CNN method provides better approximation to the original tissue echogenicity by completely removing speckle noises and improving the image resolution together with the capability for real-time implementation. Nonetheless, the limitation of the proposed CNN model is its data-dependency. For specific scenarios, it will be important to produce representative training datasets to optimize the performance of CNN model. For example, since our training data mainly comprises of cysts and edges in a uniform background, the current CNN model in this study is expected to perform better for liver imaging than for other organs. This is because the liver is a large organ composed of uniform parenchyma tissue with underlying blood vessels. Therefore, the echogenicity map of liver is structurally similar to that in the training data of this study and could be better reconstructed using the current CNN model.

Moreover, the proposed CNN model is trained under the assumption that the PSF is spatially invariant. Note that the size of PSF relies on both the transmit/receive beamforming and array geometry. In receive beamforming, it has been a common routine to adopt a constant receive f-number to alleviate the spatial variation of PSF. In transmit beamforming, the change of PSF generally comes from the effect of single transmit focusing that degrades the PSF outside the transmit focal zone. This phenomenon, however, can also be alleviated by synthetic transmit focusing to provide total focusing along the entire depth of interests such as in coherent plane-wave compounding image [25]. Even when the synthetic transmit focusing is not applicable, conventional multiple-zone focusing can still help to maintain a spatially invariant PSF in ultrasound B-mode image. For the array geometry, on the other hand, it should be noted that B-mode image using phase array and curved linear array is beamformed in polar coordinate. Thus, the lateral resolution will degrade with the imaging depth when the polar sector is converted to rectangular coordinate for image display. This is generally referred to as scan conversion. In this case, the proposed CNN model could be applied before scan conversion to suppress the speckle pattern and to restore the image resolution when the PSF remains depth-independent in polar coordinate.

**Figure 11:**
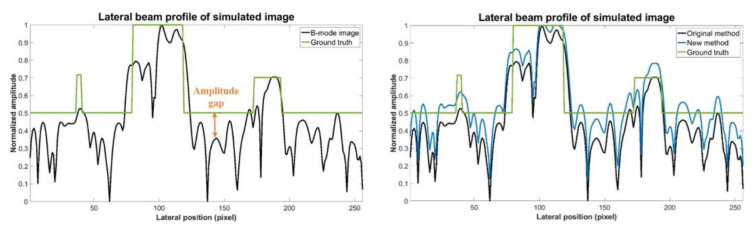
Ground truth echogenicity map and lateral profiles of the simulated B-mode images of the two normalization methods.

**Figure 12:**
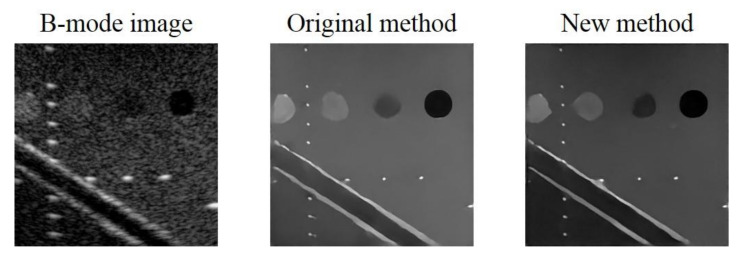
B-mode image and the reconstructed echogenicity maps of the models applied the two normalization methods.

Additionally, it should be noted that, in the preparation of training data we normalized the original echogenicity map and the simulated B-mode image by their respective maximum amplitude in the echogenicity map and the B-mode image. However, after considering Rayleigh randomization and convolving with the PSF, the difference between the maximum and minimum amplitude in the B-mode image becomes larger. Additionally, there will be an amplitude gap between the background area of the B-mode image and the ground truth echogenicity map as shown in Figure 11. Since the proposed CNN model aims to make the reconstructed echogenicity map and the ground truth as close as possible, the abovementioned amplitude gap also makes the background brightness of the reconstructed echogenicity map higher than that of the B-mode image, which leads to overestimation of the original tissue echogenicity. To overcome this problem, a new normalization method can be applied. Specifically, we use the theoretical value of the B-mode image corresponds from the maximum amplitude in the ground truth as the new normalization reference value to avoid generating excessively high maximum amplitude in the B-mode image. Figure 12 shows the reconstructed results of the proposed CNN models trained by the two normalization methods of the input B-mode image. After applying the new normalization method, the background brightness of the reconstructed echogenicity map become more similar to that of the B-mode image while the clarity of the scattering structure is still maintained. This indicates that the aforementioned overestimation of tissue echogenicity has been alleviated by using the new normalization method. Quantitative analysis of the two normalization method is listed in Table 8. Because the new normalization method will reduce the average amplitude of the background ROI, the CR and CNR both decrease. The image resolution is not much different so that the wires can still maintain high definition.

In conclusion, the proposed CNN method can effectively remove the speckle noise in the test image while maintaining the clarity of the tissue structure and improving the interpretability of the image. Since the PSF of the ultrasound imaging system actually varies with depths, we also take the factor into consideration. When estimating the PSF size of the test image to create the training dataset, we use the average value of the three wire regions with different depths as setting value. From the results, the method can achieve good speckle reduction effects at different depth in the B-mode image and reconstruct clear scattering structures. In addition, the proposed CNN method simply comprises three steps: acquire the test images from the ultrasound scanner, estimate the size of PSF and apply it to the simulated dataset for CNN training. Therefore, the proposed method can be readily generalized and applied to any other ultrasound commercial scanner to approximate original echogenicity maps of tissue.

**Figure 13:**
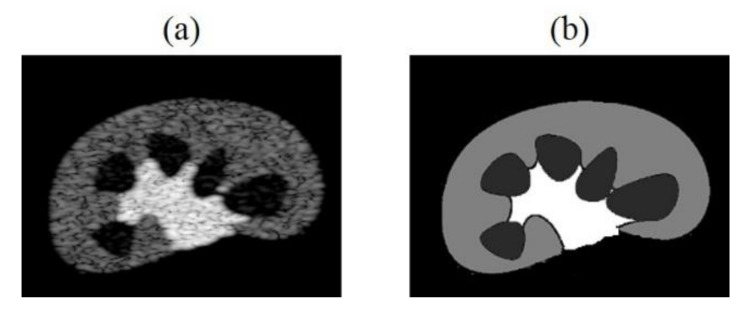
(**a**) Kidney simulation B-mode image and (**b**) echogenicity map.

In the future, we can increase the number and diversity of training dataset. For example, by producing simulated training data which contains similar structures to in-vivo imaged tissue/organ as shown in Figure 13, or making datasets that simultaneously include simulated, phantom and clinical images, raising the overall generality of models. We can apply transfer learning to initialize the network weights of a new model through the trained CNN model, speed up training convergence and increase model stability. Besides, we can also increase the training times, and make subtle adjustments of loss functions and hyperparameters of the neural network to improve accuracy of the tissue echogenicity restoration and combine it with clinical applications.

## Figures and Tables

**Table 1:** Echogenicity magnitudes of the scattering structures.

Scattering Structures	Echogenicity Magnitude (dB)
Cyst	±6, ±18, ±30, anechoic
Edge/Point	+3, +6, +12, +20

**Table 2:** Neural network setting.

Hyperparameters	Values
Initial learning rate	0.0007, 0.00035, 0.000175
Filter size	3 × 3, 5 × 5, 7 × 7
Minimum number of filters	16, 32
Epoch	30
Batch size	8

**Table 3:** Quantitative analysis of different size of PSF.

	Original	With CNN De-Speckling				
PSF size			L = 2.0 A = 2.0	L = 4.0 A = 4.0	L = 6.0 A = 6.0	L = 6.2 A = 2.8
FWHM (L/A)	5.9/2.4	MSE loss	7.6/2.1	6.4/2.2	7.4/2.2	**2.3/1.7**
		mixed loss	7.3/2.4	8.0/2.8	7.4/2.3	**2.9/2.0**
CR	0.22	MSE loss	0.28	0.29	0.29	**0.32**
		mixed loss	0.26	0.31	0.29	**0.33**
CNR	2.72	MSE loss	10.57	10.01	9.20	**25.12**
		mixed loss	12.17	20.16	10.23	**44.14**
MSE	2279.6	MSE loss	268.6	393.3	653.5	**67.9**
		mixed loss	187.6	285.9	157.3	**62.1**
SSIM	0.17	MSE loss	0.89	0.87	0.77	**0.97**
		mixed loss	0.94	0.94	0.95	**0.98**
PSNR	14.63	MSE loss	25.11	23.30	20.75	**32.07**
		mixed loss	26.50	25.08	27.61	**33.13**
MI	0.009	MSE loss	0.007	0.001	0.005	**0.015**
		mixed loss	0.003	0.004	0.002	**0.023**

**Table 4:** Quantitative analysis of different minimum number of convolutional filters.

	Original	With CNN De-Speckling	
		16 filters	32 filters
CR	0.22	0.29	**0.33**
CNR	2.72	25.13	**44.14**
FWHM(L/A)	5.9/2.4	**2.2/1.5**	2.3/1.7
MSE	2279.6	222.3	**62.1**
SSIM	0.17	0.96	**0.98**
PSNR	14.63	29.03	**33.13**
MI	0.009	0.003	**0.023**

**Table 5:** Quantitative analysis of different filter size.

	Original	With CNN De-Speckling		
		filter size 7 × 7	filter size 5 × 5	filter size 3 × 3
CR	0.22	0.28	0.32	**0.33**
CNR	2.72	25.35	33.63	**44.14**
FWHM(L/A)	5.9/2.4	3.3/2.2	**2.8**/2.2	2.9/**2.0**
MSE	2279.6	111.79	84.79	**62.1**
SSIM	0.17	0.97	0.96	**0.98**
PSNR	14.63	28.86	30.5655	**33.13**
MI	0.009	0.006	0.008	**0.023**

**Table 6:** Quantitative analysis of different initial learning rate.

	Original	With CNN De-Speckling		
		Initial lr 0.0007	initial lr 0.00035	initial lr 0.000175
CR	0.22	0.31	**0.33**	0.31
CNR	2.72	20.87	**44.14**	27.66
FWHM (L/A)	5.9/2.4	3.0/2.7	2.9/2.0	**2.6/1.6**
MSE	2279.6	109.28	**62.1**	76.90
SSIM	0.17	**0.98**	**0.98**	**0.98**
PSNR	14.63	30.64	**33.13**	32.09
MI	0.009	0.015	**0.023**	0.021

**Table 7:** Quantitative analysis of different de-speckling methods.

	Original	With De-Speckling			
Method		NLM	ADF	BLF	Proposed CNN
CR	0.22	0.21	0.22	0.21	**0.33**
CNR	2.72	5.33	3.63	5.22	**44.14**
FWHM (L/A)	5.9/2.4	6.7/2.3	5.9/2.9	7.1/3.1	**2.9/2.0**
MSE	2279.6	1702.8	2273.8	1863.2	**62.1**
SSIM	0.17	0.55	0.17	0.57	**0.98**
PSNR	14.63	15.95	14.64	15.54	**33.13**
MI	0.009	0.008	0.009	0.004	**0.023**
Execution time per image (s)		0.018	0.008	**0.007**	0.008

**Table 8:** Quantitative analysis of different normalization methods.

	Original	With CNN De-Speckling	
Normalization method		Original method	New method
CR	0.22	0.33	0.28
CNR	2.72	44.14	36.87
FWHM (L/A)	5.9/2.4	2.9/2.0	2.5/2.0
